# Extreme Sequence Divergence but Conserved Ligand-Binding Specificity in Streptococcus pyogenes M Protein

**DOI:** 10.1371/journal.ppat.0020047

**Published:** 2006-05-26

**Authors:** Jenny Persson, Bernard Beall, Sara Linse, Gunnar Lindahl

**Affiliations:** 1 Division of Medical Microbiology, Department of Laboratory Medicine, Lund University, Lund, Sweden; 2 Centers for Disease Control and Prevention, Respiratory Diseases Branch, Atlanta, Georgia, United States of America; 3 Department of Biophysical Chemistry, Chemical Center, Lund University, Lund, Sweden; University of California San Diego, United States of America

## Abstract

Many pathogenic microorganisms evade host immunity through extensive sequence variability in a protein region targeted by protective antibodies. In spite of the sequence variability, a variable region commonly retains an important ligand-binding function, reflected in the presence of a highly conserved sequence motif. Here, we analyze the limits of sequence divergence in a ligand-binding region by characterizing the hypervariable region (HVR) of Streptococcus pyogenes M protein. Our studies were focused on HVRs that bind the human complement regulator C4b-binding protein (C4BP), a ligand that confers phagocytosis resistance. A previous comparison of C4BP-binding HVRs identified residue identities that could be part of a binding motif, but the extended analysis reported here shows that no residue identities remain when additional C4BP-binding HVRs are included. Characterization of the HVR in the M22 protein indicated that two relatively conserved Leu residues are essential for C4BP binding, but these residues are probably core residues in a coiled-coil, implying that they do not directly contribute to binding. In contrast, substitution of either of two relatively conserved Glu residues, predicted to be solvent-exposed, had no effect on C4BP binding, although each of these changes had a major effect on the antigenic properties of the HVR. Together, these findings show that HVRs of M proteins have an extraordinary capacity for sequence divergence and antigenic variability while retaining a specific ligand-binding function.

## Synopsis

Many pathogens have evolved mechanisms to evade host immunity. In one such mechanism, the sequence of a surface protein varies among different strains of a pathogen. This sequence variability represents an apparent paradox, because the variable protein must retain an important function. The authors studied this problem in *Streptococcus pyogenes,* a major human pathogen. The surface-localized M protein of this bacterium varies extensively in sequence between bacterial strains, allowing immune escape. Nevertheless, the most variable part of the M protein commonly binds a human plasma protein. By hijacking this human protein the bacteria evade attack by complement an important part of the innate immune system. Comparison of the ligand-binding region in different M proteins showed that these regions lack a shared amino acid sequence motif. Thus, a variable protein can retain a ligand-binding function in the absence of a conserved binding motif. Evidence is also presented that a single amino acid change in the variable region may cause a major antigenic change, providing a selective advantage for the bacteria. Together, these data bear witness to the extraordinary ability of pathogens to escape host immunity, without losing ability to cause disease.

## Introduction

Sequence variability is a common feature in surface proteins of pathogenic microorganisms. Such variability may confer increased fitness because it allows the pathogen to use alternative receptors or allows infection of different tissues or even different species [1–5]. However, in most cases the variability probably reflects antigenic variation, which allows the pathogen to evade protective immunity in an infected host [6].

The sequence variability that gives rise to antigenic variation may be very extensive and represents an apparent paradox because the variable protein must retain an important function in spite of the variability. To explain this apparent contradiction, it is commonly assumed that conservation of a limited number of residues is sufficient to promote correct protein folding and/or to confer a specific function [7], while other residues may vary and cause changes in antigenic properties of the protein. For example, the very variable hemagglutinin of the influenza virus has a few highly conserved residues that are located in the receptor-binding pocket [8–10]. Similarly, the CD36-binding region of the Plasmodium falciparum protein PfEMP1 varies extensively in sequence, but several conserved residues were predicted to be important for binding [11]. In contrast, we show here that the hypervariable region (HVR) in streptococcal M protein, a major bacterial virulence factor, retains ability to specifically bind a human protein ligand, although different HVRs completely lack residue identities.

The Gram-positive bacterium Streptococcus pyogenes (group A streptococcus) is a major human pathogen that causes a variety of diseases, including acute pharyngitis and the streptococcal toxic shock syndrome [12]. The surface-localized M protein, which is the most extensively studied virulence factor of *S. pyogenes,* is a dimeric coiled-coil that inhibits phagocytosis and exhibits antigenic variation due to the ~50-residue N-terminal HVR [13,14]. The HVR is stable within a strain of S. pyogenes, allowing the identification of ~120 different M types [15], although limited sequence variability is occasionally observed between clinical isolates of the same M type. Thus, the number of known M types is small compared to the large number of possible sequence variants, suggesting that these M types have been selected because of their superior fitness.

**Figure 1:**
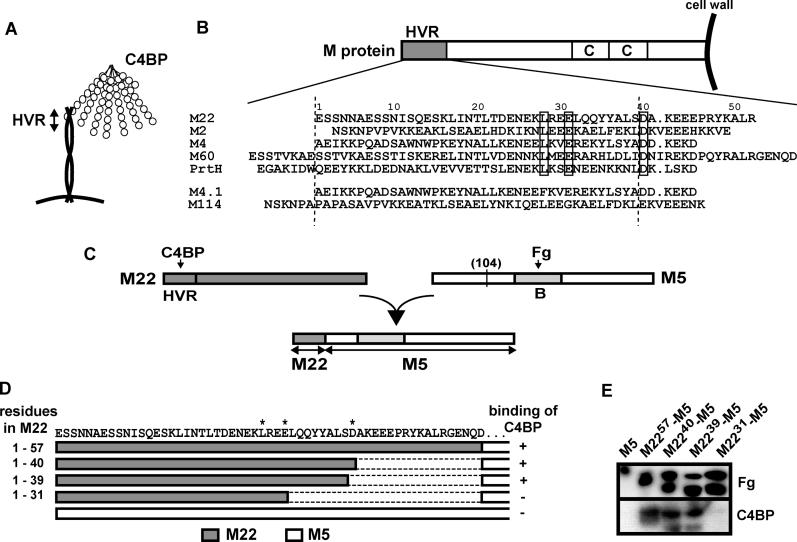
The Binding Site for Human C4BP in the Hypervariable Region (HVR) of M Protein (A) Schematic representation of C4BP bound to the HVR of an M protein, a dimeric coiled-coil. The most common form of C4BP has seven identical α-chains and one short β-chain. Both chains are composed of CCP modules, as indicated. The binding site for M protein in C4BP is located in the CCP1–2 region of the α-chain [17,24,47]. (B) Multiple sequence alignment of HVRs that bind C4BP. The five upper sequences are from [25]. Three residues that are identical in these five sequences are boxed. PrtH is a second M protein expressed by certain M1 strains [35]. The lower part of the alignment shows the HVRs of M4.1 and M114, characterized in this paper. The vertical hatched lines, corresponding to residues 1–39 in M22, indicate the region used to generate the logo in Figure 5A. (C) Construction of fusion proteins derived from the M22 and M5 proteins. An N-terminal region derived from M22 was fused to the C-terminal part of M5 (residues 104–450 of M5). The fusion proteins contain the Fg-binding B-repeat region of M5. (D) Schematic representation of the N-terminal region of different fusion proteins. The sequence of the N-terminal region of M22 is given at the top. Asterisks indicate the position of residues L28, E31, and D40 in M22 (corresponding to the three boxed residues in [B]). The ability of the fusion proteins to bind C4BP, indicated to the right, is based on the results shown in (E). (E) Ability of fusion proteins to bind C4BP. The fusion proteins (D) are referred to as M22^57^–M5, etc. Whole-cell lysates of E. coli strains, expressing the indicated proteins from genes carried on pBR322, were analyzed by Western blot using Fg or C4BP as the probe. The strain expressing M5 was used as a negative control. The control blot with Fg showed that the proteins were expressed in *E. coli.* The presence of double bands probably reflects incomplete processing of signal peptides in E. coli and/or intracellular degradation of M protein in this heterologous host.

In many M proteins, the HVR specifically binds a human complement inhibitor, the plasma protein C4b-binding protein (C4BP), which prevents complement deposition on the bacterial surface and allows the bacteria to evade phagocytosis [16–21] (Figure 1A). Because antibodies that prevent binding of C4BP promote phagocytosis [20,21], the sequence divergence among C4BP-binding HVRs probably reflects selection during evolution of antigenic variants that retain ability to bind C4BP. This argument implies that severe limitations exist on possible sequences in the HVR, a conclusion supported by extensive sequence analysis [22,23].

The C4BP-binding HVRs are distinct ligand-binding domains that bind to the same region in C4BP and probably have similar structures [18,24–26]. Nevertheless, comparison of different C4BP-binding HVRs only allowed the identification of three amino acid residue identities [18,25]. It seemed possible that these three identities were part of a binding motif, but we hypothesized that not even these residues would be required for binding of C4BP. To analyze this hypothesis we used a large collection of clinical S. pyogenes isolates and found that C4BP-binding HVRs indeed lack a common sequence motif. Thus, M proteins have an extraordinary capacity for sequence divergence while retaining the ability to specifically bind a ligand. We also present evidence that even a single amino acid change that does not affect C4BP-binding may cause a major antigenic change in an HVR, providing a molecular basis for the appearance of new M types through gradual accumulation of mutations.

## Results

### The C4BP-Binding Region in the M22 Protein

Five C4BP-binding HVRs that have been characterized previously [18,25] are aligned in the upper part of Figure 1B and the three amino acid identities in these sequences of ~50 residues are boxed. In spite of the sequence divergence, the alignment of these sequences was clear-cut, as shown by pairwise comparisons. The three identities correspond to L28, E31, and D40 in M22, an extensively studied C4BP-binding M protein which we used as model protein [18,20,21,24]. Of note, M22 is one of the most common serotypes among strains of S. pyogenes isolated in different parts of the world [27–29], making the M22 protein an attractive model protein.

A region comprising the 52 N-terminal residues in M22 is sufficient for C4BP binding [25]. To analyze whether the C-terminal part of this region, and in particular the D40 residue, is required for binding, we constructed a series of fusion proteins in which N-terminal regions of different length, derived from M22, were fused to the C-terminal part of an M protein that does not bind C4BP, the M5 protein (Figure 1C and 1D). The region derived from M5, comprising residues 104–450, had the same length in each construct and included a fibrinogen (Fg)–binding region, which was used for detection of the fusion proteins. After expression in *Escherichia coli,* the fusion proteins were analyzed by Western blot for ability to bind Fg and C4BP (Figure 1E). Constructs that included 57, 40, or 39 residues from M22 showed equally good binding of C4BP, while a construct that only included 31 residues from M22 did not bind. Of note, the ability of the M22^39^–M5 protein to bind C4BP was not due to the contribution of an Asp residue, corresponding to D40 in M22, by the M5 fusion partner because the first residue in the part derived from M5 was a Leu. These data indicate that a region comprising the 39 N-terminal amino acids in M22 is sufficient for binding of C4BP. This region only includes two of the identities, L28 and E31, with the other previously studied C4BP-binding regions (Figure 1B).

### Characterization of Additional M Proteins Demonstrates that C4BP-Binding Regions Completely Lack Residue Identities

**Figure 2:**
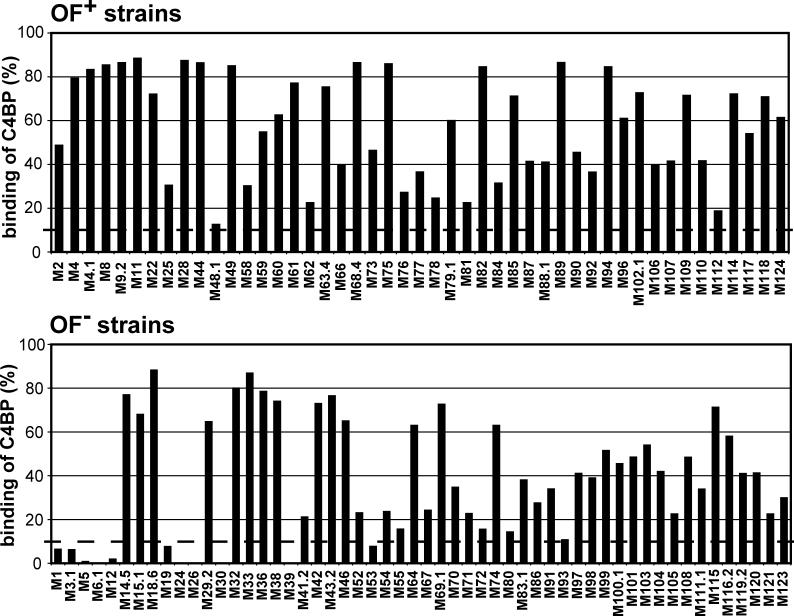
Binding of Human C4BP to S. pyogenes Strains of Different M Types Strains of the M types indicated were analyzed for ability to bind radiolabelled C4BP. Upper panel: OF^+^ strains. Lower panel: OF^−^ strains. Only strains that bound Fg, as determined in parallel tests, were used for the analysis because binding of Fg is a characteristic property of S. pyogenes isolates expressing members of the M protein family. Binding is expressed as percent of added radioactivity. The threshold for binding of C4BP was set at ≥10% binding (dashed line). Background binding to an M-negative strain (~3%) has been subtracted. All strains were tested at least twice, with duplicate samples, and the results were highly reproducible. For each strain, data from one experiment are shown. The data include binding data for one allelic variant per M type, except that data for both M4 and M4.1 are included. For strains of some M types, several allelic variants were tested and in most cases these strains did not differ in ability to bind C4BP (unpublished data).

We hypothesized that not even the two residues corresponding to L28 and E31 in M22 are conserved in all C4BP-binding M proteins. To analyze this hypothesis, we screened a large number of reference S. pyogenes strains for ability to bind C4BP and analyzed the M protein sequence in strains that were able to bind C4BP. The strains used were either opacity factor–positive (OF^+^) or OF^−^, the two major subgroups of S. pyogenes strains, and they represented most known M types and some subtypes (Figure 2). The sequence of HVRs was analyzed by using information available from epidemiological studies (http://www.cdc.gov/ncidod/biotech/strep/doc.htm).

To ensure that strains analyzed for C4BP-binding expressed M protein, they were first tested for ability to bind Fg, a characteristic property of clinical isolates expressing members of the M protein family [13,14,30]; only Fg-binding strains were analyzed for ability to bind C4BP. This step was included because a strain of S. pyogenes propagated in the laboratory may occasionally lose ability to express M protein [13]. Background binding to an M-negative strain was subtracted and the threshold for binding of C4BP was set at ≥10% binding. In this analysis, binding of C4BP was seen for all 47 OF^+^ strains studied and for 80% of the 54 OF^–^ strains (Figure 2). These results extend findings made in previous studies [16,31] and indicate that binding of C4BP is a very common property among strains of *S. pyogenes.* Interestingly, C4BP binding was observed for all tested strains of the recently recognized M types M94–M124, which include both OF^+^ and OF^−^ strains [15,32]. Based on preliminary sequence analysis of HVRs in C4BP-binding strains, our work was focused on two OF^+^ strains expressing the M114 and M4.1 proteins, respectively. The sequences of the corresponding two HVRs are aligned with the five previously studied HVRs in the lower part of Figure 1B.

The M114 protein was chosen for further study because the residue in M114 corresponding to E31 in M22 is a helix-breaking Gly, and because M114 is a common type among strains causing invasive disease (see [27], where M114 was referred to as st2967). The M4.1 protein, which is a subtype of M4, was chosen for further study because it has a Phe at the position corresponding to L28 in M22. Of note, even a conservative change from Leu to Phe may have important effects on protein structure and function, as observed for the positive gene regulator PrfA of Listeria monocytogenes [33] and the eukaryotic protein calmodulin [34].

Although it seemed likely that the HVRs in the M4.1 and M114 proteins were responsible for the ability of the corresponding strains to bind C4BP, this was not obvious because some S. pyogenes strains express a second M or M-like protein that binds C4BP. For example, some strains of serotype M1 and M18 express an M protein that does not bind C4BP, and also express an M-like C4BP-binding protein [18,35,36]. Moreover, it could not be excluded that the ability of the M4.1 and M114 strains to bind C4BP was caused by a surface structure unrelated to M proteins. Thus, it was essential to demonstrate that the HVRs of the M4.1 and M114 proteins promoted binding of C4BP.

**Figure 3:**
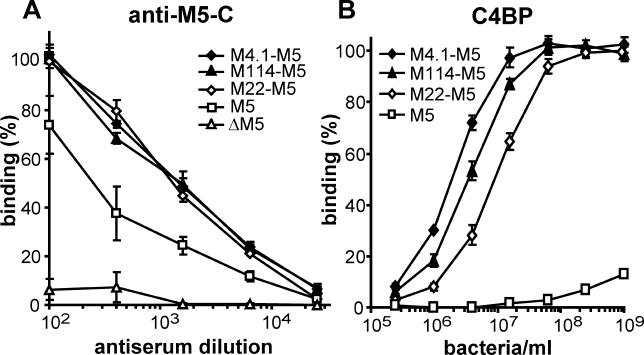
The HVRs of M4.1 and M114 Bind C4BP Fusion proteins, derived from the HVR of M4.1 or M114 and the C-terminal part of M5, were expressed in the M-negative strain S. pyogenes ΔM5 using genes carried on plasmid pLZ12Spec. Controls included a strain expressing the C4BP-binding fusion protein M22^57^–M5, a strain expressing the non–C4BP-binding M5 protein, and the M-negative strain ΔM5. (A) Surface expression analyzed with rabbit antibodies directed against the C-repeat region of M5. Bound antibodies were detected with radiolabelled protein A. Binding of protein A to the M22^57^–M5 strain, incubated with antiserum diluted ×10^2^, was defined as 100%. The M5-negative strain ΔM5 served as negative control. (B) Binding of radiolabelled C4BP. Binding at the highest bacterial concentration to the control expressing M22^57^–M5 was defined as 100%. The non–C4BP-binding M5 strain served as negative control. All data in (A) and (B) are based on three separate experiments with duplicate samples, and are presented as means ± SD.

Fusion proteins were constructed in which the HVR of M4.1 or M114 was combined with the C-terminal part of M5, generating the M4.1–M5 and M114–M5 proteins. Preliminary analysis showed that these two fusion proteins were able to bind C4BP after expression in *E. coli,* demonstrating that the HVRs of M4.1 and M114 indeed bind C4BP (unpublished data). To analyze C4BP binding in a physiological setting and to allow quantitative analysis, the two fusion proteins were characterized after expression in S. pyogenes using genes expressed from a shuttle vector in an M-negative S. pyogenes strain (Figure 3). Expression of the fusion proteins on the bacterial surface was verified by analysis with antiserum to the conserved C-repeat region of M5 (anti–M5-C). Strains expressing the C4BP-binding fusion protein M22^57^–M5 [18] or the nonbinding M5 protein served as positive controls for reactivity with the antibodies, while the M-negative strain ΔM5 served as negative control. The results (Figure 3A) show that the M4.1–M5 and M114–M5 fusion proteins were expressed on the streptococcal surface at the same level as the M22^57^–M5 protein. The somewhat lower surface expression observed for M5 might be due to a weaker promoter in the corresponding gene. When analyzed for ability to bind C4BP, the streptococcal strains expressing M4.1–M5 and M114–M5 not only showed binding, but bound C4BP even better than the control strain expressing the M22^57^–M5 protein (Figure 3B). These data show that the HVRs of the M4.1 and M114 proteins represent C4BP-binding regions similar to those previously described [18].

From the analysis of M4.1 and M114, it follows that C4BP-binding HVRs in M proteins completely lack residue identities (Figure 1B). Thus, the HVR of M protein has an extraordinary capacity to tolerate sequence divergence while retaining the ability to bind C4BP.

### The HVR of the M114 Protein Is a Distinct C4BP-Binding Domain

Previous studies with synthetic peptides, derived from the HVRs of the M2, M4, and M22 proteins, showed that these HVRs represent distinct domains that bind with high specificity to the same region in C4BP [25]. Importantly, binding of C4BP to such a peptide is strongly enhanced by peptide dimerization via a C-terminal cysteine residue [25,37]. This finding may be explained by the demonstration that the C4BP-binding HVRs probably have dimeric coiled-coil structure [26] and suggests that the coiled-coil must be stabilized by a disulphide bond in the peptides, but not in the intact M proteins. Because the HVR of M114 contains a helix-breaking Gly residue, suggesting that it might have properties different from the other HVRs, a dimerized synthetic peptide derived from this HVR was analyzed with regard to binding specificity, binding site in C4BP, and secondary structure and stability. The binding properties of the M4.1 HVR were not studied further because it was not possible to synthesize a peptide corresponding to this HVR.

**Figure 4:**
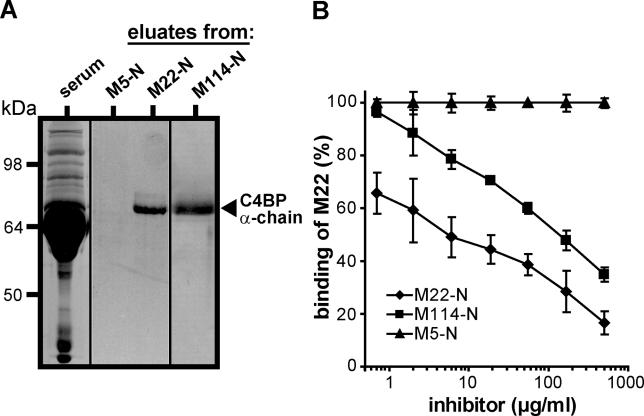
Characterization of the C4BP-Binding HVR in M114 (A) The HVR of M114 is a distinct protein domain that binds C4BP with high specificity. A dimerized synthetic peptide, derived from the 52 N-terminal residues in M114 and designated M114-N, was immobilized in a column. Whole human serum was passed through the column, which was washed and eluted. Control columns contained the C4BP-binding M22-N peptide or the nonbinding M5-N peptide. The eluates, and human serum, were analyzed by SDS-PAGE, as indicated. The ~70 kDa polypeptide present in the eluates from the M22-N and M114-N columns was identified as the C4BP α-chain by Western blot analysis with specific antiserum (not shown). (B) The M114 and M22 proteins bind to the same region in C4BP. The peptides indicated were used to inhibit the binding of radiolabelled M22 protein to C4BP immobilized in microtiter wells. Data from three separate experiments with duplicate samples, presented as means ± SD.

The synthetic peptide derived from M114, comprising the 52 N-terminal residues in the mature form of M114 and designated M114-N, was found to bind C4BP (unpublished data). The specificity of the binding was analyzed by affinity chromatography. For this purpose, whole human serum was applied to a column containing immobilized M114-N and bound protein was eluted and analyzed by SDS-PAGE [25,37] (Figure 4A). Columns containing peptides derived from the C4BP-binding HVR of M22 or the nonbinding HVR of M5 (peptides M22-N and M5-N) were used as positive and negative controls, respectively. The eluates from the M114-N and M22-N columns contained a single major polypeptide, which was identified as the C4BP α-chain, while no protein was retained on the M5-N column. Because C4BP has a serum concentration of ~200 mg/l and therefore represents <0.5% of all protein in serum [38], this result demonstrates that the M114-N peptide binds C4BP with high specificity.

**Figure 5:**
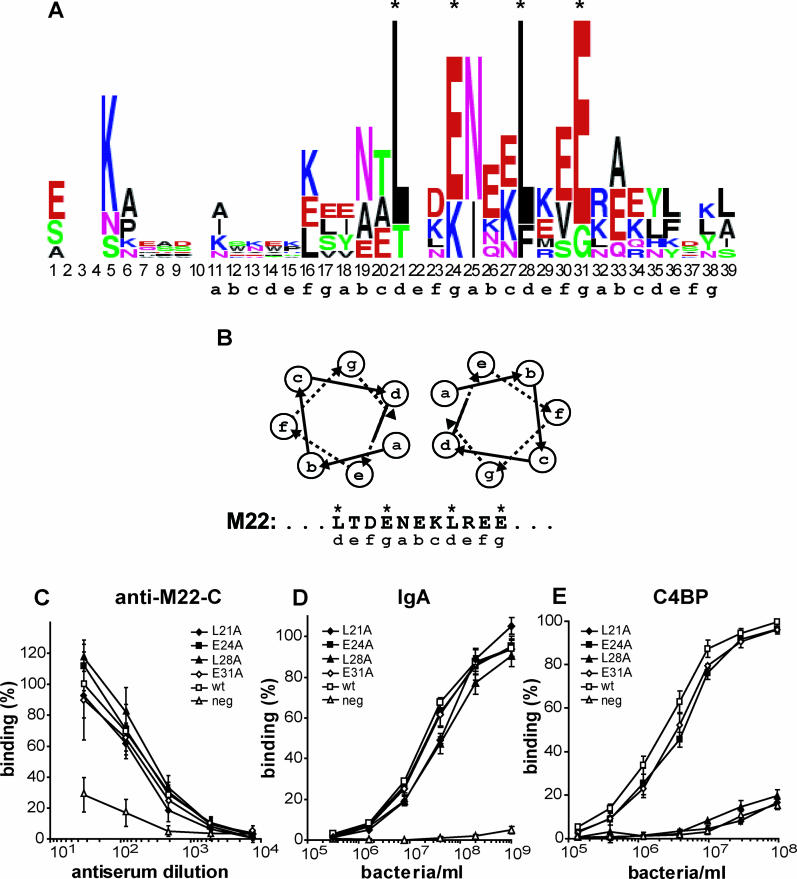
Sequence Analysis of C4BP-Binding HVRs and Site-Specific Mutagenesis in M22 (A) Sequence logo of C4BP-binding HVRs. The logo was generated from the seven C4BP-binding HVRs aligned in Figure 1B, using WebLogo (http://weblogo.berkeley.edu). Only regions in the HVRs corresponding to the shortest known C4BP-binding region in M22 (residues 1–39) were used to create the logo. These regions are demarcated by the vertical hatched lines in Figure 1B. In the logo, each column in the alignment is represented by a stack of letters, with the height of each letter proportional to the observed frequency of the corresponding residue at that position, while the overall height of each stack is proportional to the sequence conservation at that position [63]. The sequence of the M4.1 HVR was included in the generation of the logo, although it is virtually identical to M4, because the single residue difference between these two HVRs was important for the conclusion that the different HVRs completely lack residue identities (see text). The numbering below the logo refers to residue numbers in the M22 protein and putative coiled-coil heptads (a–g) in M22 are indicated. Asterisks show the position of four M22 residues (L21, E24, L28, and E31) analyzed by site-specific mutagenesis. (B) Helical wheel representation of a dimeric coiled-coil [42]. The sequence of the L21–E31 region of M22 is included, with asterisks above residues L21, E24, L28, and E31, which were analyzed by site-specific mutagenesis and are located within the predicted coiled-coil region. The positions of residues within putative coiled-coil heptads (a–g) are indicated. (C–E) The four mutant M22 proteins indicated, constructed by site-specific mutagenesis, and the wild-type (wt) M22 protein, were expressed in *S. pyogenes,* and the strains were analyzed for surface expression of the proteins and ability to bind C4BP. The genes encoding the proteins were present on plasmid pLZ12Spec, carried by an M-negative strain. This M-negative strain also served as negative control. (C) and (D) show that the different proteins were expressed normally on the bacterial surface (see text). (E) shows that mutants L21A and L28A had completely lost C4BP-binding ability, while mutants E24A and E31A were unaffected. The results shown in (C–E) are based on three separate experiments with duplicate samples and are presented as means ± SD.

To analyze whether M114 binds to the same region in C4BP as other C4BP-binding M proteins, the M114-N peptide was tested for ability to inhibit the interaction between the C4BP-binding M22 protein and immobilized C4BP (Figure 4B). The C4BP-binding M22-N peptide and the nonbinding M5-N peptide were used as positive and negative controls, respectively. The M114-N peptide inhibited the interaction, but was a less-efficient inhibitor than the M22-N peptide, possibly because M114-N binds with lower affinity. This inhibition was not unspecific because the binding of IgA to M22 [39] was not inhibited by M114-N (unpublished data). These results indicate that M114 binds to a site in C4BP that is overlapping, if not identical, with that used by M22.

Analysis of M114-N by circular dichroism spectroscopy indicated that the secondary structure of this peptide is similar to that of M4-N and M22-N (unpublished data). Moreover, the melting temperature of M114-N was lower than for M4-N and M22-N, possibly reflecting lower stability of this peptide because of the presence of the helix-breaking Gly residue (unpublished data).

A previous immunological analysis of C4BP-binding synthetic peptides derived from the HVRs in M2, M4, and M22 showed that they lack cross-reactivity, although they have similar binding properties [25]. In agreement with these findings, M114-N was not recognized by antibodies to the HVRs of the M4 and M22 proteins, but showed limited cross-reactivity with the HVR of the M2 protein, to which M114-N is most closely related (unpublished data). These results confirm that C4BP-binding HVRs show great antigenic variability.

Together, the analysis of M114-N indicates that this peptide has properties similar to other peptides derived from C4BP-binding HVRs in spite of the presence of a Gly residue in M114-N.

### Sequence Analysis and Site-Specific Mutagenesis

Although the seven C4BP-binding HVRs described above exhibit extreme sequence divergence (Figure 1B), a sequence logo of these HVRs shows that the region comprising residues 21–31 (numbering based on M22) is less variable than the other parts of the HVRs (Figure 5A). This is not surprising because some residues may be expected to be more important than other ones for structure and/or ligand binding, although no single residue is absolutely required for ability to bind C4BP.

The conserved parts of M proteins are known to be dimeric coiled-coils [40,41], but it has remained unclear whether the HVRs also form coiled-coils. Indeed, a previous computational analysis suggested that the HVRs may adopt helix-turn-helix conformation [25]. However, the recent nuclear magnetic resonance study by André et al. [26] indicates that the HVRs do have coiled-coil conformation. This situation made it of interest to analyze whether the distribution of residues in the alignment region represented in the logo was compatible with coiled-coil structure in the HVRs. A coiled-coil is characterized by a seven-residue periodicity in which the residues are designated a–g. Residues at positions a and d most often are hydrophobic and constitute the core of the coiled-coil, while the other residues are solvent-exposed [42] (Figure 5B). However, in M or M-like proteins, the heptad patterns often show a nonoptimal distribution of residues [41,43], and in some M proteins position a is commonly occupied by an Asn residue [40]. The distribution of amino acid residues over the aligned region fits well with the hypothesis that the less variable region corresponding to residues 21–31 in M22 is part of a coiled-coil.

We used site-specific mutagenesis of M22 to analyze the role of different residues for C4BP binding. These studies were focused on the four relatively conserved residues L21, E24, L28, and E31, which are located within the predicted coiled-coil region (Figure 5A and 5B). The L21 and L28 residues are predicted to be core residues that occupy position d in the coiled-coil, while residues E24 and E31 are predicted to be solvent-exposed residues occupying position g. Each of these residues was changed to Ala and the four mutant M22 proteins were expressed in *S. pyogenes.* To analyze whether the mutant proteins were expressed normally on the bacterial surface, the strains were analyzed for reactivity with antibodies against the conserved C-repeat region in M22 and for ability to bind human IgA, which specifically binds to M22 [39]. The analysis with anti-C serum was performed with antibodies raised in the rat, because rabbit antibodies show Fc-reactivity with M22 [39]. Analysis with this rat serum showed that the mutant proteins were present on the streptococcal surface in the same amounts as the wild-type protein expressed by the positive control (Figure 5C), and similar results were obtained in binding analysis with IgA (Figure 5D). Thus, the mutant M22 proteins were expressed normally on the streptococcal surface, making them suitable for analysis of the role of the mutated residues in binding of C4BP (Figure 5E).

The L21A and L28A mutants had completely lost ability to bind C4BP, a finding that may be explained by the key role that residues in heptad position d play as core residues in a coiled-coil. In contrast, the E24A and E31A mutants were not affected in ability to bind C4BP, indicating that the corresponding residues are not essential for binding of C4BP although they probably are surface-exposed and are relatively conserved among the sequences studied here.

The sequence logo in Figure 5A was derived from seven HVRs known to bind C4BP (Figure 1B). This analysis was supported by a logo derived from a larger number of HVRs, which are not definitely known to bind C4BP but probably do so (Figure S1A). This logo was similar to that derived from the known C4BP-binding HVRs (Figure 5A), indicating that the observed pattern may reflect an inherent property of C4BP-binding HVRs. In contrast, a logo derived from 11 non–C4BP-binding HVRs had another appearance (Figure S1B), suggesting that the distribution of residues is different for those HVRs that do not bind C4BP.

### Single Amino Acid Changes in M22 that Do Not Affect C4BP Binding Cause Major Immunological Changes

The sequence variability in the HVR of M proteins causes antigenic variation, allowing a strain expressing one M protein to escape recognition by antibodies directed against other M proteins [13]. The simplest explanation for this sequence variability is that it has arisen through gradual accumulation of mutations, each of which causes a change in antigenicity and at least partial escape from host immunity (antigenic drift). It may appear intuitively obvious that antigenic variants must be selected through this mechanism, but it is not clear how the change of a single amino acid residue can alter the antigenic properties of a protein to such an extent that it can escape a polyclonal antibody response (i.e., antibodies that probably recognize multiple epitopes). Indeed, there is only little evidence for this hypothesis in the literature [44,45]. We used the M22 system to analyze this problem.

**Figure 6:**
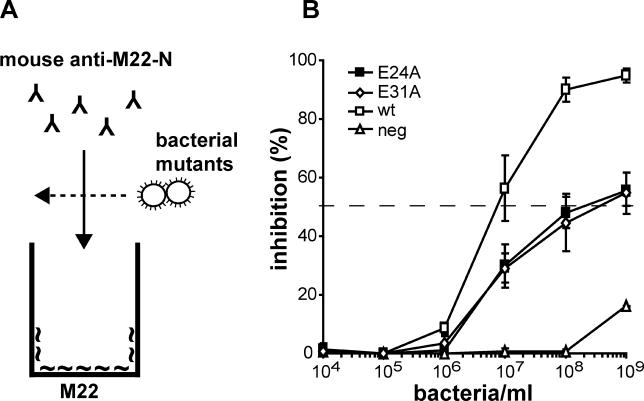
Single Amino Acid Changes Not Affecting C4BP Binding Cause Major Antigenic Changes in the HVR of M22 (A) Schematic representation of an inhibition test used to analyze the antigenic properties of mutant M22 proteins expressed on the surface of *S. pyogenes.* The test was based on the binding of mouse anti–M22-N to pure M22 protein immobilized in microtiter wells. This binding was inhibited with whole S. pyogenes bacteria expressing mutant M22 proteins. (B) Ability of S. pyogenes strains expressing the M22 mutants E24A and E31A to cause inhibition. The positive control expressed the wild-type M22 protein and the negative control lacked M protein. As compared to the positive control, 50% inhibition (dashed line) required ~30-fold more bacteria expressing either of the mutant proteins. Results based on three separate experiments with duplicate samples, presented as means ± SD.

As shown in Figure 5E, the two changes E24A and E31A had no effect on the ability of the surface-expressed M22 protein to bind C4BP. This finding made it of interest to analyze the antigenic properties of the mutants. For this purpose, we used an inhibition test (Figure 6A). Purified M22 protein was immobilized in microtiter wells and detected with polyclonal mouse serum raised against the M22-N peptide (i.e., the HVR of M22). Mouse serum was used because the M22 protein binds to the Fc part of rabbit IgG but does not bind mouse antibodies in such nonimmune fashion [39]. To analyze the effect of the E24A and E31A mutations on the antigenicity of the HVR in M22, we used whole streptococci expressing the mutant proteins to inhibit binding of the mouse antibodies to immobilized M22. This experimental procedure allowed comparison of the antigenic properties of different M22 proteins expressed on the streptococcal surface (i.e., under physiological conditions). Control bacteria expressed the wild-type M22 protein or no M protein. Interestingly, a 50% reduction in binding required ~30-fold more bacteria expressing either of the mutant proteins, as compared to bacteria expressing wild-type M22 protein (Figure 6B). This result was not due to reduced surface exposure of the mutant proteins (Figure 5C and 5D). Thus, the single amino acid changes E24A and E31A, which do not affect the ability of M22 to bind C4BP, cause major changes in the immunological properties of the protein.

## Discussion

In many strains of *S. pyogenes,* the binding of human C4BP to M protein plays an important role for the ability of the bacteria to evade phagocytosis [20,21]. This binding is highly specific in spite of the sequence variability among C4BP-binding HVRs. Indeed, the HVRs only bind C4BP among all human plasma proteins [25,37], and C4BP only binds to M or M-like proteins among all S. pyogenes surface proteins [18,21,36,46]. Moreover, the only human protein that binds to the same region in C4BP as M protein is the natural ligand C4b [17,24]. Here, we have shown that the C4BP-binding HVRs completely lack residue identities (i.e., they do not share any conserved sequence motif). However, a sequence alignment indicated that the C-terminal half of the HVRs is more conserved than the N-terminal half, suggesting that this part is of particular importance for binding because interaction with the ligand may pose restraints on variability. The hypothesis that the relatively conserved region, corresponding to L21–E31 in M22, is part of the binding region is supported by inhibition experiments with short synthetic peptides in the M4 system [18]. However, it is difficult to make predictions about the role of different residues in this region for the binding of C4BP, because no residue is completely conserved.

A recent nuclear magnetic resonance study indicates that a major part of a C4BP-binding HVR has coiled-coil structure and the C4BP-binding site was localized to a region corresponding to residues 13–39 in M22 [26]. However, it has not yet been possible to conclusively determine the structure of an HVR, so the available data must be interpreted with caution. Our sequence analysis and mutagenesis data support the coiled-coil model for the HVR because replacement of the relatively conserved L21 or L28 residues in M22 with Ala completely abolished C4BP binding. Although it cannot be excluded that these Leu residues are directly involved in binding, this result can most easily be explained by distortion of a coiled-coil structure. In contrast, replacement of the relatively conserved E24 and E31 residues (which are predicted to be surface-exposed in a coiled-coil) with Ala did not have any apparent effect on C4BP binding. Moreover, the position occupied by E31 in M22 is occupied by a helix-breaking Gly residue in M114. Thus, it is not clear why E24 and E31 are relatively conserved among the C4BP-binding M proteins. Further analysis of the role of different residues in the HVR will require determination of the structure of one or more HVRs in complex with C4BP.

While the predicted coiled-coil structure of the HVR still represents a model, the structure of the M protein-binding region in C4BP has recently been determined [47]. Moreover, residues in C4BP implicated in binding of the M4 protein were identified [47]. The binding region in C4BP is located in the α chain, in which the first two complement control protein (CCP) modules are necessary and sufficient for binding [17,24,47]. The M protein–binding site is most likely located at or near the intermodular interface and in a patch on CCP2 [47]. Electrostatic interactions play a role in binding, but other forces probably also contribute, as witnessed by the lack of dependence on salt and pH [24,47]. Thus, much information is available concerning the interaction between C4BP and M protein, but these data do not provide an explanation for the ability of C4BP to specifically bind HVRs with very different sequences.

The extreme sequence divergence in the C4BP-binding HVR of M protein contrasts with some well-known systems, such as the hemagglutinin of the influenza virus, which exhibits extensive sequence variability but nevertheless retains some completely conserved residues that are required for ligand binding [8–10]. Even the very variable gp120 protein of HIV-1 contains some highly conserved residues, which have been implicated in binding to the cellular receptor CD4 [48–50]. These comparisons raise the question why the C4BP-binding HVRs exhibit such extensive sequence divergence.

One possible explanation for the remarkable sequence divergence in C4BP-binding HVRs is that M proteins have been under stronger selective pressure for change than most other surface proteins in pathogens, including the two viral proteins mentioned above. However, it is difficult to envisage how selective pressure from the immune system could have caused greater variability in S. pyogenes than in rapidly mutating RNA viruses such as influenza virus or HIV-1. An alternative explanation is that the C4BP-binding HVRs employ a binding mechanism that easily permits sequence variability. According to one interesting hypothesis, main-chain atoms in the HVR make an important contribution to the binding surface, a situation that would make the interaction at least partially independent of amino acid sequence. Binding of C4BP via main-chain atoms may also explain why the HVRs have very different antigenic properties, although they probably have similar structure, because antibodies preferentially contact side chains [51]. Thus, C4BP and antibodies may bind to the HVR by different mechanisms. Precedence for binding via main-chain atoms in a variable region comes from studies of gp120 in HIV-1, in which half of the residues that contact human CD4 do so only through main-chain atoms [49]. Moreover, studies of Pseudomonas aeruginosa pilin suggest that the receptor-binding surface may be dominated by main-chain atoms that interact with a disaccharide on target cells [52].

Another mechanism that may contribute to the ability of HVRs with very different sequences to bind C4BP could involve the many charged residues in the HVRs. Although the combined effect of these charged residues may be important for binding, it is conceivable that the HVRs behave as if they were saturated with charge, making them insensitive to a single-charge substitution, as described for peptides binding the eukaryotic protein calmodulin [53].

The appearance of antigenic variation through antigenic drift implies that a single amino acid change may alter the antigenic properties of a protein to such an extent that it can at least partially escape a polyclonal antibody response. There is only little evidence for this important hypothesis, but our analysis of M22 variants, constructed by site-specific mutagenesis, demonstrated that single amino acid changes, which did not affect C4BP-binding, indeed caused a major change in the antigenic properties of the protein. Although the mutations analyzed here have not been identified among clinical S. pyogenes isolates, these results support the notion that novel antigenic types may appear through gradual accumulation of single amino acid changes. However, the mechanism by which a single amino acid change may cause a major alteration in antigenic properties, without affecting ligand-binding properties, remains unclear. One explanation for this remarkable situation could be that a single residue change indirectly affects the structure of all surface epitopes without affecting the ligand-binding site [44]. Alternatively, a polyclonal antibody response may be composed of a limited antibody repertoire, allowing escape of immune attack also through a limited structural change [54].

In summary, comparison of seven M proteins shows that their C4BP-binding HVRs completely lack residue identities, although they specifically bind to the same region in C4BP. This sequence divergence represents a striking example of Darwinian evolution in a microbial surface protein, which varies to evade immune attack in infected hosts but simultaneously must retain an important function [55]. Such extreme sequence divergence may occur also in other ligand-binding virulence factors that are major targets for host immunity, and it underlines the difficulty in identifying conserved sequence motifs suitable for vaccine development. Finally, the work described here is of interest for structural biology, because it implies that microbial protein regions lacking residue identities may adopt the same structure, allowing them to specifically bind the same ligand.

## Materials and Methods

### Bacterial strains, plasmids, and culture conditions.

The S. pyogenes strains expressing the M5, M12, M22, and M60 proteins have been described [16,18]. All other wild-type S. pyogenes strains were reference strains from the Center for Disease Control and Prevention, Atlanta, GA. On the basis of the M protein expressed by these strains, they are referred to here as M2, M4, etc., except that some of the isolates represented allelic variants and are designated M3.1, M14.5, etc. The M-negative S. pyogenes mutants ΔM5, derived from strain M5 Manfredo, and AL168*mrp^−^emm^−^,* derived from the reference M22 strain AL168, have been described [46,56]. The E. coli strains LE392, KJ622 [57], or TG1 were used for subcloning.

Plasmid pBR322 carries a gene for ampicillin-resistance. Plasmid pKEJ1 is a derivative of pBR322 carrying the *emm*5 gene with a restriction site for BglII at nucleotide 474 [18]. Plasmid pLZ12Spec is an *E. coli–S. pyogenes* shuttle vector carrying a spectinomycin resistance gene [58]. A derivative of pLZ12Spec carrying the *emm*22 gene has been described [56].

E. coli strains were cultured in Luria-Bertani broth. S. pyogenes strains were grown in Todd-Hewitt broth supplemented with 0.2% yeast extract and incubated without shaking in 5% CO_2_ at 37 °C. Strains of E. coli carrying derivatives of pBR322 were grown in the presence of ampicillin (100 μg/ml). Strains carrying pLZ12Spec were grown in the presence of spectinomycin (20 μg/ml for E. coli and 70 μg/ml for S. pyogenes).

### Fusion proteins.

In the M4.1–M5 and M114–M5 fusion proteins, the region comprising the first 45 amino acid residues of M4.1, or the first 53 residues of M114, is fused to residues 104–450 of M5. For construction of genes encoding these proteins, the promoter region and the region encoding the indicated N-terminal region of M4.1 or M114 was amplified by PCR using chromosomal streptococcal DNA as template. The DNA fragments were ligated into plasmid pKEJ1 digested with SalI and BglII. A similar procedure was used to prepare constructs encoding the fusion proteins M22^31^–M5 and M22^40^–M5. The construct encoding M22^39^–M5 was derived from that encoding M22^40^–M5 by using the QuickChange mutagenesis kit (Stratagene, La Jolla, California, United States). The construct encoding M22^57^–M5 has been described [18]. The sequence of all clones was confirmed by DNA sequencing. For expression of fusion genes in *S. pyogenes,* they were transferred to pLZ12Spec, followed by transformation into the M-negative S. pyogenes strain ΔM5. Because all fusion proteins studied here included the Fg-binding B-repeat region and the C-repeat region of M5, the proteins could be identified by ability to bind Fg or antiserum to the C repeats.

### Site-specific mutagenesis.

Site-specific mutagenesis was performed according to Berggård et al. (2001). The procedure employed an XhoI site and an Mph1031 site in the *emm*22 gene, located at positions corresponding to amino acids S8–N10 and Y36–L38, respectively. The Mph1031 site was present in the wild-type *emm*22 gene, while the XhoI site had been introduced by site-specific mutagenesis and caused an amino acid change. However, this XhoI site was eliminated in the final construct (see below). To introduce a mutation in the region between the two restriction sites, a plasmid carrying the *emm*22 gene (with the XhoI and Mph1031 sites) was digested with XhoI and Mph1031, followed by replacement of the deleted fragment with a synthetic linker containing the desired sequence change. The linker was constructed to destroy the XhoI site, thereby restoring the wild-type sequence at that site. Due to difficulties during the cloning work described here, the *emm*22 gene subjected to mutagenesis was not carried on plasmid pLZ12Spec, as previously described [20], but was transferred to pBR322. After ligation of the linkers into the cleaved plasmid, the construct was transformed into E. coli LE392 and clones were screened for presence of the substitution by XhoI digestion of PCR products. Clones that were negative in this screening were analyzed for protein expression in E. coli and verified by DNA sequencing. The mutated *emm*22 genes, lacking the XhoI site, were transferred back to pLZ12Spec to allow transformation into streptococci. This procedure was used for construction of the mutated genes encoding the E24A, L28A, and E31A proteins. The gene encoding the L21A protein was generated by PCR on plasmid pLZ12Spec carrying the *emm*22 gene using the QuickChange site-specific mutagenesis kit. This change introduced a restriction site for AlwNI, a property used to screen PCR products. Positive clones were confirmed by DNA sequencing. The pLZ12Spec derivatives encoding M22 mutant proteins were transferred into the M-negative S. pyogenes strain AL168*mrp^−^emm^−^*.

### Purified proteins and synthetic peptides.

The M22 (Sir22) protein was purified as described [39]. Human C4BP was purified as described [37]. Human Fg was from American Diagnostica (Stamford, Connecticut, United States) and human serum IgA was from Cappel Organon-Teknika (Turnhout, Belgium). Staphylococcal protein A and streptococcal protein G were from Amersham Biosciences (Uppsala, Sweden). The synthetic peptides M5–N and M22–N were derived from the N-terminal 50 or 52 amino acid residues of the mature forms of M5 and M22, respectively [25]. The M114-N peptide was derived from the N-terminal 52 residues of the predicted mature form of the M114 protein. These peptides were purchased from The Department of Clinical Chemistry, Malmö General Hospital, Lund University (Malmö, Sweden). Each of the M5-N, M22-N, and M114-N peptides included a C-terminal cysteine residue, not present in the intact M protein, to allow dimerization via a disulphide bridge [25]. Dimerization was performed as described [25].

### Antisera.

Rabbit antiserum against a peptide derived from the C-repeat region in M5 (anti–M5-C) was prepared as described [25]. Rat antiserum against a synthetic peptide derived from the C-repeat region of M4/M22 and designated anti–M22-C was prepared as described [59]. Antiserum against the M22-N peptide was raised in mice [21]. Rabbit anti-mouse immunoglobulins were from DakoCytomation (Glostrup, Denmark).

### Binding tests and inhibition tests.

Binding of radiolabelled human Fg, IgA, or C4BP to whole streptococci was analyzed as described [46]. In brief, streptococci from overnight cultures were washed in PBS supplemented with 0.02% NaN_3_ and 0.05% Tween 20 (PBSAT) and resuspended to a concentration of 10^9^ bacteria/ml. The streptococci were diluted as indicated in a suspension of E. coli (to provide a pellet in subsequent centrifugation steps) and incubated with radiolabelled ligand (~14,000 cpm) at room temperature for 1 h. After washes, the radioactivity associated with each pellet was measured in a gamma counter. To screen many S. pyogenes strains for ability to bind C4BP (Figure 2), radiolabelled C4BP was incubated with duplicate samples (200 μl) of bacterial suspensions containing 10^9^ bacteria/ml. Each strain was analyzed at least twice with similar results**.** Background binding to an M-negative strain (~3%) has been subtracted.

Binding of rat or rabbit antibodies to whole streptococci was analyzed essentially as described [60]. Briefly, washed overnight cultures of streptococci in PBSAT were diluted to 10^9^ bacteria/ml and samples (200 μl) were incubated for 1 h at room temperature with rat or rabbit antiserum diluted in PBSAT. For detection of bound antibodies, the washed bacteria were incubated with radiolabelled protein A or protein G (~14,000 cpm in 200 μl). After washing, radioactivity associated with each pellet was measured in a gamma counter.

Inhibition tests with the M22-N and M114-N peptides (Figure 4B) were performed essentially as described [25]. Briefly, human C4BP was immobilized in microtiter wells, which were blocked with PBSAT, and radiolabelled M22 protein (~14,000 cpm/well) was added together with a solution of unlabelled peptide to achieve a final concentration of 0–500 μg/ml. After incubation for 1 h at room temperature, the wells were washed and radioactivity associated with each well was determined.

The ability of whole streptococci to inhibit binding between pure M22 and mouse anti-M22-N (Figure 6) was performed essentially as described [21]. In brief, suspensions of whole washed bacteria, diluted as indicated, were prepared in mouse anti–M22-N (diluted 200-fold), and added to microtiter wells coated with M22 protein (50 μl, 1 μg/ml). After washings (to remove bacteria and mouse antibodies not bound to the immobilized M22), mouse antibodies bound to the immobilized M22 protein were detected with rabbit anti-mouse Ig and radiolabelled protein G. A control with preimmune mouse serum did not show any binding to M22, showing that the binding of the anti–M22-N serum was specific. Of note, mouse C4BP does not bind to M22 and should not affect the analysis [17,37].

### Affinity chromatography.

Chromatography of human serum using immobilized peptides was performed essentially as described [25,37]. Briefly, 5 mg of dimerized peptide (M5-N, M22-N, or M114-N) was immobilized in a 1 ml HiTrap column containing *N*-hydroxysuccinimide–activated agarose beads (Amersham Pharmacia Biotech, Little Chalfont, United Kingdom). Human serum (5 ml) diluted 5-fold in TBS (20 mM Tris, 0.15 M NaCl [pH 7.4]) was applied to the column, and after ten washes with TBS (1 ml), bound proteins were eluted**,** dialyzed against TBS, and analyzed by SDS-PAGE.

### Sequence analysis.

A multiple sequence alignment of C4BP-binding HVRs (Figure 1B, upper part) was constructed with the CLUSTALW [61] algorithm with the BLOSUM62 [62] residue substitution score matrices. The shortest known C4BP-binding region of each protein, as determined with fusion proteins or synthetic peptides [18,25], was included in the alignment. Note that the sequence of the M4 HVR shown here is the wild-type sequence and differs from the sequence in the previously characterized M4-N peptide at one position because the R32 residue was substituted for a Lys in the peptide for technical reasons [25]. The C4BP-binding HVRs of the M4.1 and M114 proteins (Figure 1B, lower part) were aligned manually to the other sequences. The sequence logos in Figure 5A and Figure S1 were generated using WebLogo (http://weblogo.berkeley.edu) [63]. A coiled-coil prediction (Figure 5A) was generated using the COILS algorithm [64].

### Other methods.

Radiolabelling of proteins with carrier-free ^125^I (Amersham Pharmacia Biotech) was performed with the chloramine-T method [65] or a modified lactoperoxidase method [66].

## Supporting Information

Figure S1 Sequence Logos Derived from HVRs in M Proteins The sequence logo in Figure 5A was derived from seven HVRs known to bind C4BP (Figure 1B). To analyze additional C4BP-binding HVRs, we compared the HVRs in M proteins of all OF^+^ strains studied here. Although molecular analysis has not conclusively shown that these HVRs bind C4BP, it seems likely that they do because all OF^+^ strains bind C4BP (Figure 2, upper panel), and because the ability to bind C4BP has been attributed to the M protein HVR in all OF^+^ strains analyzed [18,25] (this paper). To analyze non–C4BP-binding HVRs, we used data for the 11 nonbinding strains included in Figure 2, lower panel.
(A) Logo derived from the HVRs in 47 M proteins expressed by OF^+^ C4BP-binding strains of different serotype (i.e., all strains in upper panel of Figure 2). This logo is similar to that derived from known C4BP-binding HVRs (Figure 5A). In particular, the C-terminal half is less variable than the N-terminal half and includes two dominating Leu residues and a preponderance of negatively charged residues.
(B) Logo derived from 11 non–C4BP-binding HVRs. The appearance of this logo is different from that of the logos in Figures 5A and (A). Although dominating Leu residues are seen also in this logo (most likely reflecting a coiled-coil structure), the variability is similar in both halves of the logo, and it is not clear that the C-terminal half contains a preponderance of negatively charged residues. The logos must be compared with caution, but this analysis suggests that the distribution of residues is different for those HVRs that bind C4BP and those that do not.
To construct these logos, residues 1–50 of the indicated HVRs were aligned using ClustalW. The two most conserved Leu residues were used to manually align these HVRs to those analyzed in Figure 5A. Note that the logos shown here only include the 39 residues predicted to correspond to the C4BP-binding region analyzed in Figure 5A.
(303 KB PDF)

### Accession Numbers

The GenBank (http://www.ncbi.nlm.nih.gov/Genbank) accession numbers for the genes and gene products discussed in this paper are C4BP α-chain (M31452), M2 (EmmL2.1) (X61276), M4 (Arp4) (X15198), M22 (Sir22) (X75750), M60(Arp60) (Z22751), and PrtH (M29398). Sequences for the HVRs of the M4.1 and M114 proteins, and sequences for M protein HVRs of the strains analyzed in Figure 2, are available at the Centers for Disease Control *Streptococcus pyogenes emm* sequence database (http://www.cdc.gov/ncidod/biotech/strep/doc.htm).

## Abbreviations

C4BP: C4b-binding protein
CCP: complement control protein
Fg: fibrinogen
HVR: hypervariable region
OF: opacity factor
