# Supplementary figures and images for: Extreme Sequence Divergence but Conserved Ligand-Binding Specificity in Streptococcus pyogenes M Protein
Source: PLoS Pathog. 2006 May 26;2(5):e47. doi: 10.1371/journal.ppat.0020047 (PMC1464397; doi:10.1371/journal.ppat.0020047)

Figure S1

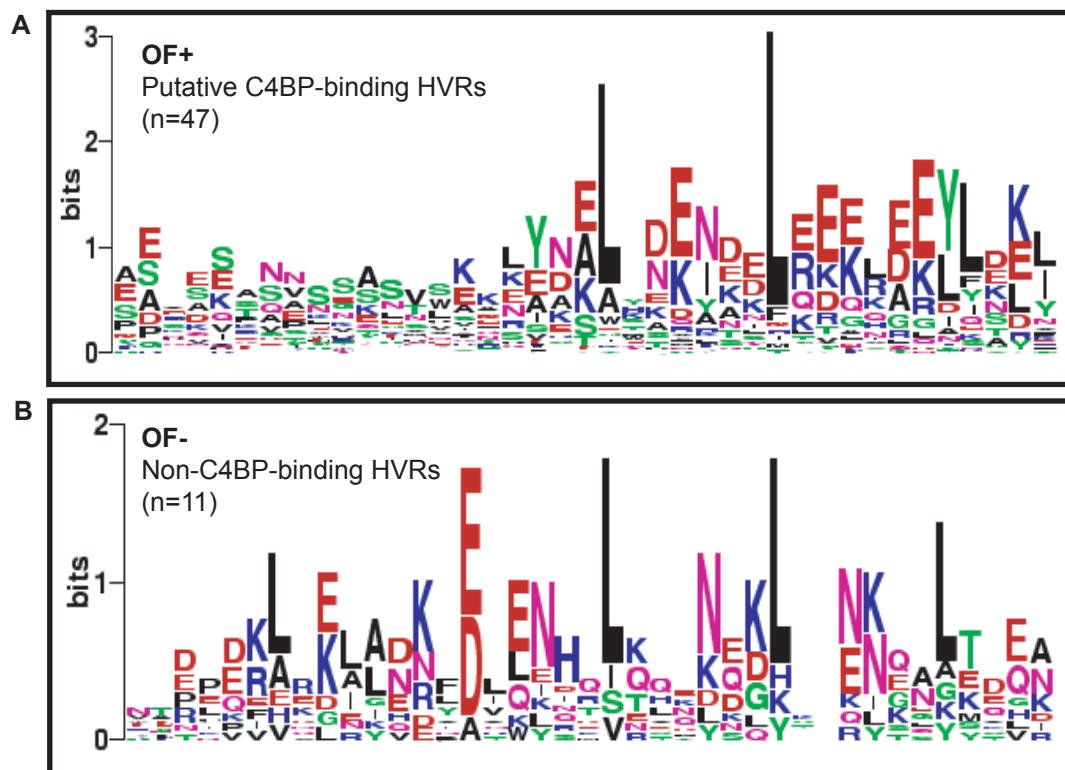

Supplement: Figure S1 — The sequence logo in Figure 5A was derived from seven HVRs known to bind C4BP (Figure 1B). To analyze additional C4BP-binding HVRs, we compared the HVRs in M proteins of all OF+ strains studied here. Although molecular analysis has not conclusively shown that these HVRs bind C4BP, it seems likely that they do because all OF+ strains bind C4BP (Figure 2, upper panel), and because the ability to bind C4BP has been attributed to the M protein HVR in all OF+ strains analyzed [18,25] (this paper). To analyze non–C4BP-binding HVRs, we used data for the 11 nonbinding strains included in Figure 2, lower panel. (A) Logo derived from the HVRs in 47 M proteins expressed by OF+ C4BP-binding strains of different serotype (i.e., all strains in upper panel of Figure 2). This logo is similar to that derived from known C4BP-binding HVRs (Figure 5A). In particular, the C-terminal half is less variable than the N-terminal half and includes two dominating Leu residues and a preponderance of negatively charged residues. (B) Logo derived from 11 non–C4BP-binding HVRs. The appearance of this logo is different from that of the logos in Figures 5A and (A). Although dominating Leu residues are seen also in this logo (most likely reflecting a coiled-coil structure), the variability is similar in both halves of the logo, and it is not clear that the C-terminal half contains a preponderance of negatively charged residues. The logos must be compared with caution, but this analysis suggests that the distribution of residues is different for those HVRs that bind C4BP and those that do not. To construct these logos, residues 1–50 of the indicated HVRs were aligned using ClustalW. The two most conserved Leu residues were used to manually align these HVRs to those analyzed in Figure 5A. Note that the logos shown here only include the 39 residues predicted to correspond to the C4BP-binding region analyzed in Figure 5A. (303 KB PDF) [file ppat.0020047.sg001.pdf]
